# Corrigendum: Repeatability and Reproducibility of *in-vivo* Brain Temperature Measurements

**DOI:** 10.3389/fnhum.2021.780797

**Published:** 2021-11-26

**Authors:** Ayushe A. Sharma, Rodolphe Nenert, Christina Mueller, Andrew A. Maudsley, Jarred W. Younger, Jerzy P. Szaflarski

**Affiliations:** ^1^Department of Psychology, University of Alabama at Birmingham (UAB), Birmingham, AL, United States; ^2^Department of Neurobiology, University of Alabama at Birmingham (UAB), Birmingham, AL, United States; ^3^University of Alabama at Birmingham Epilepsy Center (UABEC), Birmingham, AL, United States; ^4^Department of Neurology, University of Alabama at Birmingham (UAB), Birmingham, AL, United States; ^5^Department of Radiology, Miller School of Medicine, University of Miami, Miami, FL, United States; ^6^Department of Neurosurgery, University of Alabama at Birmingham (UAB), Birmingham, AL, United States

**Keywords:** MRS, brain temperature, MR thermometry, neuroinflammation, neuroimaging

In the original article, there was a mistake in ***Figure 1*** as published.

The original figure was adapted from Dehkharghani et al. (2015), but this adaptation was not appropriately described and referenced in the manuscript. We apologize for this oversight. The figure has been revised: the adapted portion has been replaced with a new graphic and the caption now appropriately indicates that a portion was adapted from a previously published article. The corrected *Figure 1* appears below.

**Figure 1:**
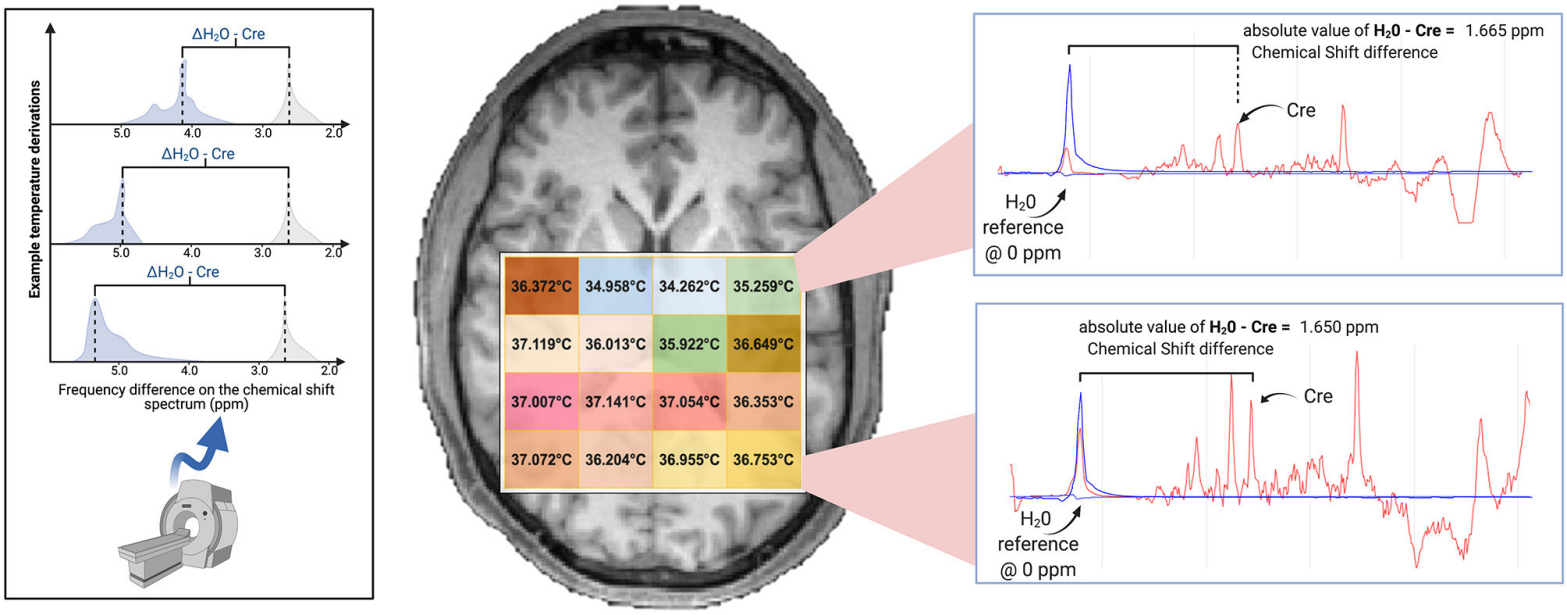
Brain temperature can be non-invasively derived from volumetric magnetic resonance spectroscopic imaging (MRSI) data by calculating the frequency difference between the temperature-sensitive water peak and one or more metabolite peaks that are temperature-insensitive (left^*^). When using creatine as the reference, voxel-level brain temperature can be calculated according to the following equation: T_CRE_ = −102.61(Δ_H20−CRE_) + 206.1°C, Δ_H20−CRE_ = chemical shift difference between the creatine and water resonances. Example T_CRE_ calculations are provided for a participant's single tissue slice (*right*). Representative spectra illustrate Δ_H20−CRE_ derivations, with plots depicting a water-suppressed metabolite spectrum (*red line*), with an overlay that indicates the location of the reference water signal (*blue line)*. Spectral plots were created within the Metabolite Imaging and Data Analysis System (MIDAS) software package, and the figure was created using BioRender. ^*^Adapted from Dehkharghani et al. (2015).

The authors apologize for this error and state that this does not change the scientific conclusions of the article in any way. The original article has been updated.

## Publisher's Note

All claims expressed in this article are solely those of the authors and do not necessarily represent those of their affiliated organizations, or those of the publisher, the editors and the reviewers. Any product that may be evaluated in this article, or claim that may be made by its manufacturer, is not guaranteed or endorsed by the publisher.
